# Postprandial Glycemia and Insulinemia Responses to a Standard and Modified Muffin in Healthy Adults and Adults with Type 2 Diabetes

**DOI:** 10.3390/foods14244318

**Published:** 2025-12-15

**Authors:** Justen T. Stoner, Alex Buga, Christopher D. Crabtree, Bradley T. Robinson, Drew D. Decker, Teryn N. Sapper, Madison L. Kackley, Christopher T. Simons, Ken Lee, Jeff S. Volek

**Affiliations:** 1 Department of Human Sciences, The Ohio State University, Columbus, OH 43210, USA; stoner.160@buckeyemail.osu.edu (J.T.S.);; 2 Department of Radiology, The Ohio State University, Columbus, OH 43210, USA; 3 Department of Food Science and Technology, The Ohio State University, Columbus, OH 43210, USA

**Keywords:** product testing, postprandial glucose, type 2 diabetes, kinetics, low-carbohydrate

## Abstract

**Background/Objectives:** Modifying standard foods to minimize postprandial glucose (PPG) and insulin excursions may benefit health. We conducted a randomized, double-blind, crossover study to compare postprandial responses to an isocaloric (~250 kcal) Standard Muffin (SM) containing refined flour and sugar versus a Modified Muffin (MM) consisting of wheat-free grains and low sugar levels in healthy individuals and individuals with type 2 diabetes (T2D). **Subjects/Methods:** Nineteen subjects (ten healthy, nine with T2D) participated in the trial. PPG responses were measured in capillary blood over 5 h to calculate the change in glucose from the baseline (ΔGLU), peak glucose (GLU_Peak_), peak–nadir glucose (GLU_P-N_), and 2 h incremental area under the curve (iAUC). Plasma insulin responses were quantified via assays. **Results:** Compared to the SM, the MM significantly reduced the GLU_Peak_ (55%), GLU_P-N_ (35%), and 2 h iAUC (80%). Within the cohort, the MM elicited a 68% reduction in the GLU_Peak_ response compared to the SM in individuals with T2D. Insulin was lower following the MM (−62%). Participants reported similar subjective taste and mouthfeel ratings. **Conclusion:** A novel MM decreased PPG and insulin responses compared to a SM, without compromising taste, thus highlighting salient features for T2D nutrition management.

## 1. Introduction

A growing body of evidence suggests that short-term glycemic responses to standardized meal challenges (i.e., postprandial glucose; PPG) can predict the onset of prediabetes beyond fasting blood glucose alone [1] and often in the absence of glycated hemoglobin (HbA1c) results [2]. If sustained over time, the repeated consumption of sugar-containing food products, specifically desserts, can have untoward effects on the area under the curve for glycemia, insulinemia, and HbA1c—all comprising the cluster of etiologies underlying the early onset of metabolic syndrome and T2D [3].

In a 20-year follow-up study, researchers determined that Level 1 hyperglycemic responses (blood glucose: 181–250 mg/dL) [4] are more closely tied to all-cause and cardiovascular death than fasting blood glucose (FBG) [5]. Moreover, emerging evidence further suggests that the 2 h PPG response is more strongly correlated with cardiovascular sequalae than HbA1c [6], specifically myocardial infarction events [7], further highlighting the importance of managing diurnal glucose patterns over fasting metrics alone. Flattening the PPG responses is thus an attractive, non-pharmacological target for novel food items [8], particularly with carbohydrate restrictions [9].

Replacing dietary sucrose and its derivatives with artificial sweeteners has been a reliable food innovation strategy to reduce the glycemic index (GI) and, consequently, PPG iAUC [10]. Specifically, products that contain resistant starch/fiber [11,12,13,14,15,16] and non-nutritive sweeteners [17,18,19,20,21] can collectively lower PPG responses by between 33 and 55%. Despite the growing popularity among consumers, there are two major considerations that need to be reconciled: cost and effectiveness. Recent work has demonstrated that minimally modified muffins are equally comparable in price to standard sugar muffin products, whereas further ingredient modifications (i.e., adding more fiber or non-nutritive ingredients) could plausibly inflate the alternative muffin food price for the consumer, albeit well within reasonable price ranges [14]. The increased demand for low-GI products and the food science innovations in alternative ingredient manufacturing thus position novel bakery products as a cheap and evidence-based solution for managing glycemia across all adults, especially in adults with impaired glucose tolerance.

The primary objective of this study was to compare the PPG responses between a Standard Muffin, made with carbohydrates from refined flour and sugar, versus a Modified Muffin, made with wheat-free grains and alternative sweetener products, in both adults with and without diabetes. Secondly, we assessed hormonal responses and perceptual sensory evaluations in response to each product as a function of health status (healthy vs. T2D). We hypothesized that the Modified Muffin product would lower PPG responses and insulin concentrations and elicit similar sensory evaluations compared to the standard product.

## 2. Materials and Methods

### 2.1. Study Participants

The Institutional Review Board (IRB) at Ohio State University approved all protocols and consent forms (IRB#2022H0438; 25 January 2023) prior to subject enrollment. All subjects signed an informed consent document prior to participation. The investigation was conducted in accordance with the principles outlined in the Declaration of Helsinki (1975, revised in 2013). All test sessions were performed at the Ohio State University Department of Human Sciences.

Nineteen participants (ten healthy controls, nine with T2D) were recruited through word of mouth, ResearchMatch databases, and emails sent to Food Science and Technology list servers routinely used for sensory science studies. Interested subjects attended an informational session that included assessment of height, weight, HbA1c, and fasting blood glucose (FBG). They also completed a medical, exercise and nutrition history questionnaire. Adults between 30 and 50 years of age who were either nondiabetic (HbA1c < 5.7%) or had a diagnosis of T2D by a health care provider and/or a current HbA1c ≥ 6.5% were eligible [22]. Both physician diagnosis of T2D and/or HbA1c status were utilized, as most individuals are immediately prescribed glucose and HbA1c-lowering medications upon T2D diagnosis. Exclusion criteria included the following: known allergies to any of the study’s product ingredients, active respiratory infection, consuming of more than 3 alcoholic drinks per day or 18 alcoholic drinks per week, and pregnancy or active breastfeeding.

### 2.2. Experimental Design

**Figure 1:**
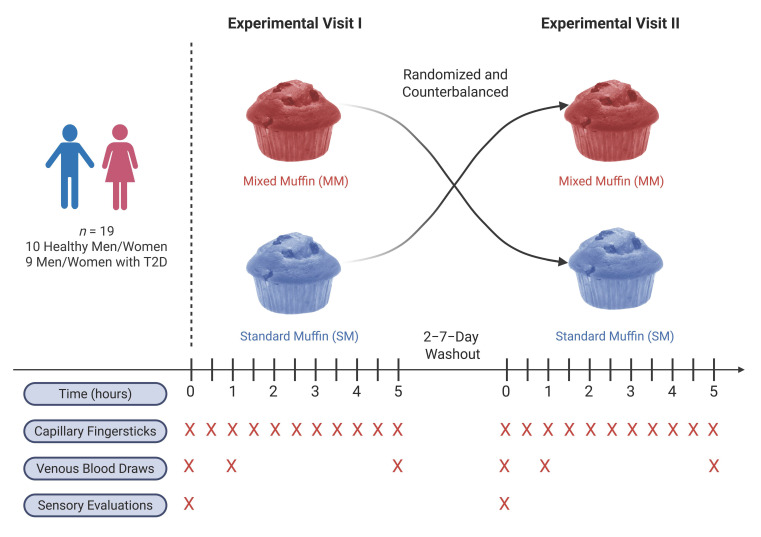
Experimental Design. Nineteen adults—ten healthy and nine diagnosed with T2D—received one full serving of a MM and SM in random and crossover fashion. Capillary blood glucose responses were measured every 30 min following product consumption via fingersticks. Venous blood draws were performed at baseline and 1 h and 5 h post-consumption. Sensory evaluations were completed immediately after product consumption. The red “X” denotes the instances when measures were collected. T2D = type 2 diabetes. Created in BioRender. Buga, A. (2025) https://BioRender.com/6jn32ka (accessed on 9 December 2025).

This was a double-blind, crossover study that compared postprandial responses after consumption of two commercially available muffins in two groups of adults who were either nondiabetic or had a diagnosis of T2D (Figure 1). Participants completed two test visits where they consumed the two muffin test products in a randomized counter-balanced manner. The visits were separated by a 2–7 day-washout period.

### 2.3. Testing Visits

All participants reported to the testing facility fasted (≥10 h), hydrated (urine specific gravity ≤ 1.025), and having abstained from consuming alcohol or performing intense physical activity more than 24 h prior to their visits to minimize confounding effects on glucose metabolism. Upon arrival, weight and height were measured on an electronic stadiometer (SECA 305, Hamburg, Germany) to the nearest 0.1 kg and centimeter, respectively. After baseline and fasting blood measurements, participants were instructed to consume the entire muffin test product. Participants were provided with water as needed to assist in swallowing and digestion. Sensory evaluations of the blinded muffin product were assessed immediately after product consumption. Participants remained in a resting state for the full 5 h period. Capillary glucose was measured every 30 min following muffin consumption. Venous blood draws were collected at 1 h and 5 h post-consumption. Each participant completed a 2–7-day washout period and then crossed over in a random counter-balanced manner to the other muffin product condition for their second experimental visit.

### 2.4. Blood Testing

Capillary blood glucose concentrations were assessed using a portable monitoring device and reagent testing strips (Keto-Mojo, Napa, CA, USA). Blood was collected by fingerstick using a lancet after the fingertip was cleaned with alcohol. The first droplet was wiped away with a cotton pad, and subsequent blood droplets were used to analyze capillary glucose concentrations. Capillary glycated hemoglobin was measured via a portable HbA1c analyzer (PTS Diagnostics A1Cnow, Whitestown, IN, USA).

A phlebotomist collected blood via venipuncture in the antecubital fossa using 21G butterfly needles and a 10 mL ethylene diamine tetra acetate (EDTA) vacuum tube (Eppendorf, Hamburg, Germany). Tubes were inverted 8 times and centrifuged immediately at 2000× *g* for 10 min at 4 °C to obtain plasma. Plasma samples were aliquoted into individual storage tubes and snap-frozen in liquid nitrogen. These aliquots were stored at −80 °C for future analysis and thawed a single time for analysis of hormones.

Insulin and leptin were analyzed in duplicate using a U-PLEX Human Custom Metabolic Group Assay kit (Meso Scale Discovery, Rockville, MD, USA). Plates were coated, sealed, and incubated for 1 h while shaking at 700 rpm at room temperature. Plates were then washed three times with 150 µL PBS-T wash solution before 50 µL of calibrator was added to each well. Plates were again sealed and incubated for 2 h while shaking at 700 rpm at room temperature. Plates were washed again with the identical protocol before adding 50 µL of 1× Detection Antibody Blend Solution in each well. Incubation occurred for 1 h while shaking at 700 rpm at room temperature. The plates were washed again before adding 150 µL of MSD Gold Read Buffer B to each well. The plates were then read on the MESO QuickPlex SQ 120 MM. The duplicate samples’ within- and between-plate coefficients of variance were 3.8% and 8.5%, respectively. The homeostasis model assessment of insulin resistance (HOMA-IR) was calculated from fasting glucose and insulin using established standards [23].

### 2.5. Muffin Products

The two muffins tested—SM (Duncan Hines, Conagra Brands, Chicago, IL) and MM (TruEats, Arora Food Group LLC, Flower Mound, TX, USA)—are both commercially available and require standard preparation methods including mixing the dry muffin contents with eggs, water, and oil. The total energy content of the muffin mix, including the contribution from eggs and oil that were added during the cooking process, are summarized in the table below (Table 1). The nutritional fact labels, cooking instructions, and manufacturer ingredient lists are included in the Supplementary Materials. The products were stored in a commercial-grade freezer with individual serving sizes being thawed overnight prior to a testing session and served at room temperature. The different muffin products were isocaloric and had similar macronutrient distribution, with the primary difference being the source of carbohydrates. The SM contained 17 g of sugar and processed wheat flour, whereas the MM had 1 g sugar and a mix of various starches from chickpea, almond, buckwheat, moong dal, and urad dal flours. Compared to SM, MM product was lower in sodium (−110 mg), saturated fat (−2.5 g), total carbohydrates (−3 g), and net carbohydrates (−19 g) and higher in erythritol (+14 g), fiber (+2 g), and protein (+2 g). Two MMs were provided to participants compared to one SM, to be as isocaloric as possible.

### 2.6. Sensory Evaluation

A 6-item product acceptability questionnaire that assesses overall liking, flavor liking, mouthfeel liking, flavor strength, sweetness, and mouth-drying sensation was administered immediately after consumption in each muffin condition. Overall liking, flavor liking, and mouthfeel liking items were answered after the initial bite of muffin product and were assessed on a 9-point scale spanning from “dislike extremely” to “like extremely” [24]. A score of 1 represented “dislike extremely”, while a score of 9 represented “like extremely.” Flavor strength, sweetness, and mouth-drying sensations were assessed after ingestion of the entire product and were assessed on a 5-point Just-About-Right (JAR) scale spanning from “much too weak” to “much too strong” [25]. From 1 to 5, each score represented “much too weak,” “too weak,” “just about right,” “too strong,” and “much too strong.”

### 2.7. Statistical Analysis

Analyses were performed using SPSS Statistics version 29 (IBM Corp., Armonk, NY, USA) and GraphPad Prism 9 (GraphPad, San Diego, CA, USA), with two-tail α significance set at *p* ≤ 0.05. Participant characteristics at baseline between nondiabetics and those with T2D were compared using an independent t-test. Sphericity was determined using Mauchly’s sphericity test and addressed with the Greenhouse–Geisser epsilon correction when necessary. We used a 2 (product) × 2 (group) × 11 (timepoints) repeated measures analysis of variance (RM ANOVA) to compare peak glucose amplitude from baseline (ΔGLU). A 2 (product) × 2 (group) RM ANOVA was used to compare peak normalized glucose (GLU_peak_), which utilized the peak glucose value across all timepoints, and peak–nadir glucose amplitude (GLU_P-N_), which utilized the difference between GLU_peak_ and the lowest capillary glucose value after peak. Hormones were statistically analyzed using a 2 (product) × 2 (group) × 3 (timepoints) RM ANOVA to best capture hormone responses [26]. Overall sensory outcomes between products were compared using an independent t-test. Incremental area under the curve (iAUC) was calculated using the trapezoidal method, where only the positive baseline-subtracted values were included in the iAUC calculation [27]. All significant post hoc effects were inspected using the Bonferroni-corrected method for multiplicity.

## 3. Results

### 3.1. Baseline Characteristics

The two groups were matched for age but differed in metabolic status, with the T2D group weighing more and having significantly higher FBG, fasting plasma insulin (FPI), HOMA-IR, and HbA1c values (Table 2). The average HbA1c in the T2D group was 5.7%, indicative of a prediabetic status, reflecting that this group was relatively well-managed, presumably due, in part, to the prescription of glucose-lowering medications in most individuals in this group.

### 3.2. Capillary Glucose Responses

**Figure 2:**
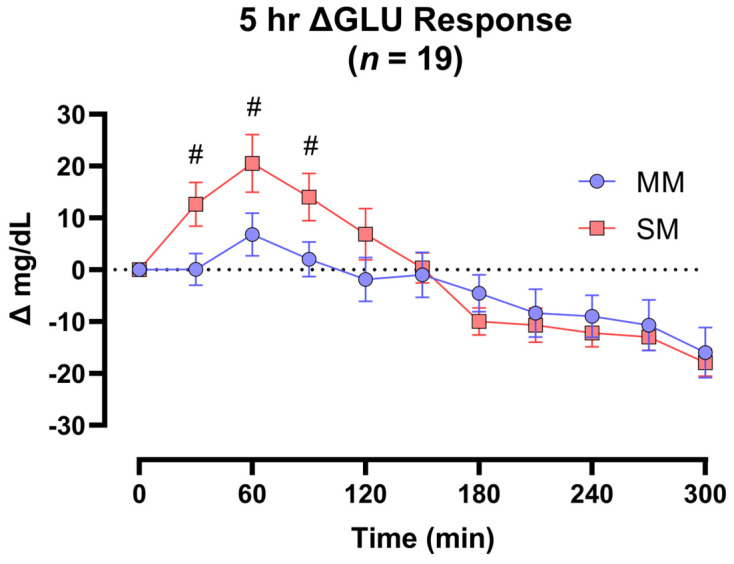
The 5 h postprandial glucose responses. There were no significant differences in ΔGLU responses between Healthy and T2D cohorts. When the cohorts were analyzed together, the significant condition interaction (product × time) revealed that the MM product significantly reduced the normalized PPG responses relative to the SM at 30 (−13.0 mg/dL), 60 (−14.3 mg/dL), and 90 (−12.6 mg/dL) min post-ingestion. Datapoints are displayed as mean ± SEM. ^#^ *p* < 0.05.

There was a main effect of time (*p* < 0.001), but no group (*p* = 0.758) or product effects were observed (*p* = 0.344) for ΔGLU. A significant ΔGLU product × time (*p* < 0.001) interaction revealed significantly different ΔGLU values between the muffin products at 30 min (*p* = 0.016), 60 min (*p* = 0.027), and 90 min (*p* = 0.017), irrespective of diabetes status (*n* = 19) (Figure 2). A trend for product × group × time interaction effects (*p* = 0.070) was observed for ΔGLU comparisons between healthy and T2D groups. The post hoc analysis revealed that significant ΔGLU differences between muffin products were present at 30 min (*p* = 0.005), 60 min (*p* = 0.008), and 90 min (*p* = 0.002) in the T2D group, resulting in an average ΔGLU response of 12.8 mg/dL, smaller at these three timepoints following the MM consumption compared to the SM.

**Figure 3:**
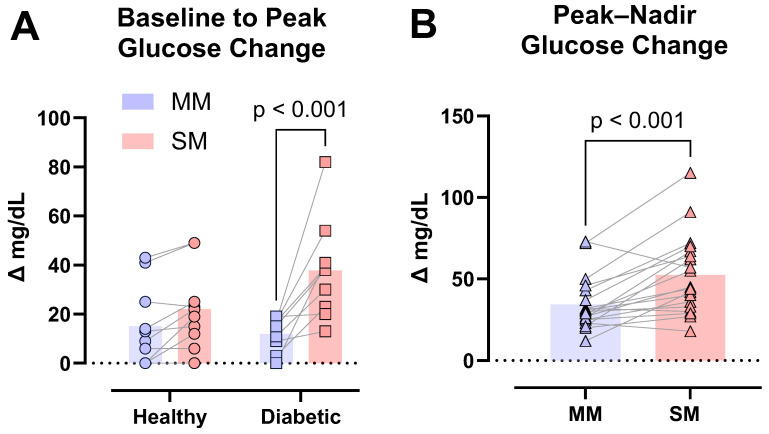
Peak glucose (GLU_peak_) and peak–nadir glucose (GLU_P-N_) responses. GLU_peak_ was calculated by subtracting the baseline glucose reading from the maximum observed glucose reading (i.e., normalized difference). GLU_P-N_ was calculated by subtracting the minimum observed glucose reading from the maximum (i.e., min–max calculation). Post hoc analysis revealed significantly different GLU_peak_ responses between muffin products in the T2D group (*p* < 0.001) (**A**). Differences (*p* < 0.001) between MM and SM products were observed in whole-group analysis (*n* = 19) (**B**).

There was a main effect of product (*p* < 0.001) but no group effects (*p* = 0.342), while a significant GLU_Peak_ product × group interaction (*p* = 0.011) was observed. The post hoc analysis revealed an average GLU_Peak_ response 68% lower following the MM consumption vs. SM consumption in the T2D group (*p* < 0.001) but not in the healthy group (*p* = 0.295) (Figure 3A).

There was a main effect of product for GLU_P-N_ (*p* < 0.001), but no group main effect (*p* = 0.079) was observed. The MM consumption resulted in a 35% decrease in the GLU_P-N_ response compared to the SM (Figure 3B). No product × group interaction effect (*p* = 0.315) was observed for GLU_P-N_.

**Figure 4:**
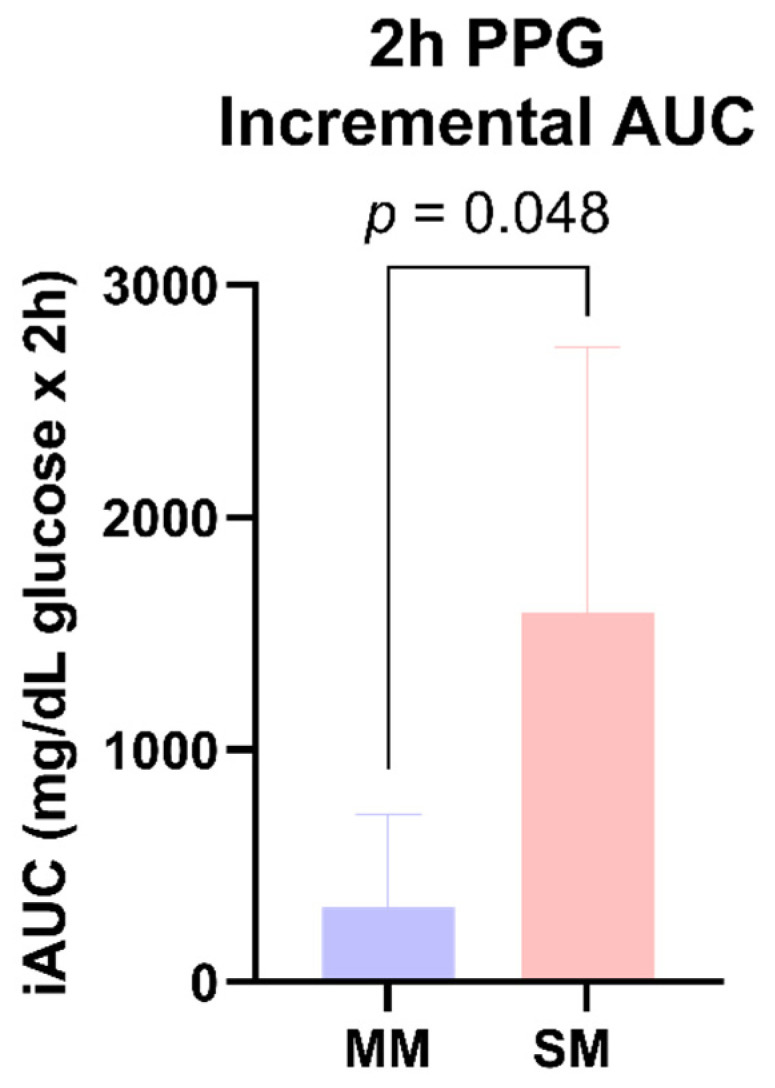
The 2 h postprandial glucose iAUC responses.

There was a main effect of the product for the 2 h PPG iAUC (*p* = 0.048). The MM and SM products elicited iAUCs of 320.7 mg/dL and 1589.0 mg/dL × 2 h, respectively, over the 2 h measurement period, independent of health status (Figure 4). There were no significant main effects by group (*p* = 0.411) or product × group interactions (*p* = 0.087).

The test battery was abbreviated to a 2 h post-ingestion window to examine the largest incremental glycemia responses. There were no significant postprandial (PP) glycemic responses between the healthy and diabetic cohorts within the 2 h iAUC window; however, the MM product produced a significantly smaller iAUC response compared to the SM, independent of the cohort health status (−80%; *p* = 0.048), denoting greater PPG control.

### 3.3. Blood Hormone Responses

**Figure 5:**
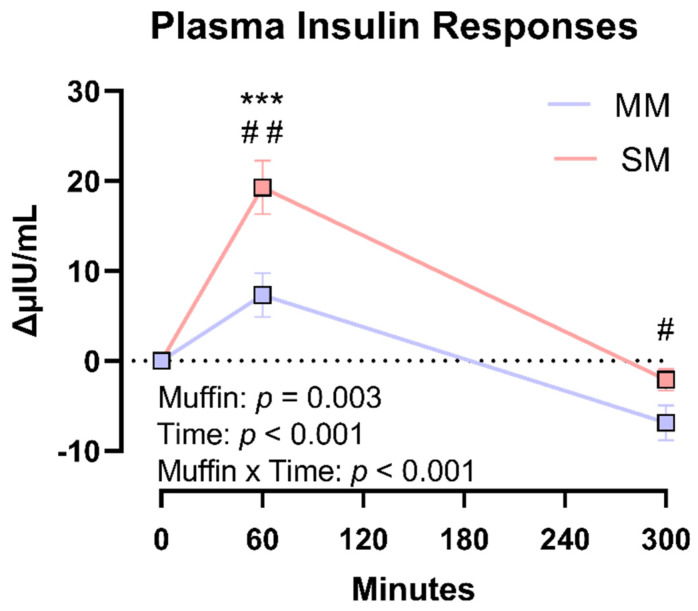
Plasma insulin responses. There were significant differences in postprandial plasma insulin responses between MM and SM products. The significant condition interaction (product × time) revealed that the MM product significantly reduced the plasma insulin response at 60 min post-consumption. Datapoints are displayed as mean ± SEM. Main effects: time, *** *p* < 0.001 compared to 0 min, and interaction, # *p* < 0.05 and ## *p* < 0.005 at indicated times.

Plasma insulin produced significant main effects for the time (*p* < 0.001) and product (*p* = 0.003) and a time × product interaction (*p* < 0.001). Post hoc analyses for the interaction effect revealed a 62% reduction in insulin responses at 60 min (*p* = 0.004) following the MM consumption compared to the SM consumption (Figure 5). A lack of main group effects (*p* = 0.847) and product × group × time interaction effects (*p* = 0.526) prevented statistical comparisons between metabolic cohorts for insulin responses.

No main effects for the time, group, or product were observed in the leptin analysis. A Pearson correlation analysis across the entire cohort showed a strong, positive relationship between fasting leptin levels and BMI (r = 0.525, *p* = 0.021), with no significant correlation between the leptin response at 60 min or 300 min and BMI status.

### 3.4. Sensory Evaluations

There were no differences in the perceptual sensory evaluation between muffin products within and between healthy and T2D cohorts (Table 3).

## 4. Discussion

This study examined whether a MM lowered postprandial glucose and hormonal responses compared to a SM in both nondiabetic adults and an age-matched cohort with T2D. The MM, which included wheat-free grains and noncaloric alternative sweeteners, decreased PPG and insulin responses in both groups, with no difference between muffins in sensory ratings. The frequent inclusion of foods that elicit lower PPG and insulin levels into meal plans without compromising flavor and taste could be helpful in the long-term management of glucose-intolerant and insulin-resistant conditions.

The consumption of the MM versus the SM decreased the total daily hyperglycemia level and could perhaps contribute to a reversal of the propensity for developing T2D [28]. Nearly all participants experienced lower GLU_Peak_ following the MM consumption compared to the SM, which is congruent with ΔGLU changes and further confirms the consistent ability of the MM to lower PPG responses independent of the starting levels of the FBG, an important outcome due to the increasing prevalence of prediabetes [29] and T2D [30]. Overall amplitude changes, measured through GLU_P-N_, were 35% lower after the MM than the SM, suggesting an improvement in the rate of the glucose uptake by peripheral tissue [31]. The improvements in the GLU_Peak_ and GLU_P-N_ suggest that, compared to the SM, the MM produces a more favorable postprandial insulin response for both healthy and T2D individuals, which is confirmed by the overall 2.6-fold reduction in insulin responses at 60 min following the MM consumption. The 2 h PPG iAUC was nearly 4-fold lower following the MM compared to the SM consumption. The 4-fold improvement in glucose iAUC shows major reductions in total glucose exposure following the MM consumption. This is important as individuals with elevated glucose iAUC responses have shown reduced β-cell function and insulin sensitivity [32], and the glucose iAUC is used as a measure for predicting the development of T2D in previously healthy individuals [33]. Based on our cross-sectional findings, replacing SMs with MMs could plausibly confer better PPG control and reduce the need for anti-hyperglycemic medication, making it an attractive dessert option without compromising taste. Longitudinal trials that assess this effect over multiple feeding instances with a greater glycemic resolution (i.e., continuous glucose monitors) are warranted.

While many results for MMs versus SMs were similar between nondiabetic and T2D cohorts, the T2D cohort demonstrated a greater mean ΔGLU reduction of 24 mg/dL across 30 min, 60 min, and 90 min after the MM compared to the SM consumption. This amplified benefit of the MM may simply reflect the higher fasting glucose and absolute PPG concentrations in the T2D group, but this indicates a benefit of lowering PPG and HbA1c in individuals with T2D [34]. There was a 68% lower GLU_peak_ after the MM than the SM in the T2D group, which was not evident in the healthy nondiabetic group. If sustained over time, the improvements in the ΔGLU and GLU_peak_ after the MM may facilitate improvements in β-cell function [35], improve insulin sensitivity and lipid metabolism, and decrease the risk of developing coronary heart disease [36].

Despite isocaloric servings, the lower PPG and insulin responses following the MM highlight the effectiveness of non-nutritive and modified ingredients. Approximately half of the total carbohydrates in the MM was in the form of a sugar alcohol, erythritol (14 g out of a total 27 g of carbohydrates), compared to the use of sucrose in the SM. Erythritol has no energy and a minimal impact on blood glucose and insulin levels [37]. The MM also consisted of alternative, gluten- and wheat-free flours, including chickpea flour, almond flour, and buckwheat flour, which are all modified flours that have reliably shown improvements in PPG responses compared to traditional whole wheat, gluten-containing flours [38,39,40]. Furthermore, the MM product tested herein included moong dal flour and urad dal flour. To our knowledge, the effects of these flours have not been reported. Although the individual effects of each alternative flour type are difficult to parse given the mixed macronutrient nature of the MM, we can reasonably infer that moong dal flour and urad dal flour have either no impact or further beneficial effects on PPG levels.

The inter-individual variability in blood glucose responses to each muffin product warrants further investigation into personalized nutrition. Regarding the GLU_peak_ and GLU_P-N_, 16% of individuals (3/19) experienced a reverse effect, where the SM consumption resulted in a minimized or identical glucose response compared to the MM consumption, highlighting the concept that the MM consumption appears not to be effective at improving postprandial responses across all individuals. While notable, this reverse effect was likely seen in our study as 3/3 individual GLU_peak_ and 2/3 individual GLU_P-N_ cases presented elevated FBG values prior to the MM consumption compared to the SM consumption, artificially increasing GLU_peak_ and GLU_P-N_ responses. Additionally, the reverse effects seen in the three individual cases were minimal, as the GLUpeak was on average 0.6 mg/dL larger, and GLUP-N was 7.6 mg/dL larger in the MM condition.

The comparable mouthfeel sensory responses between the SM and MM suggest that healthy or T2D adults can simultaneously enjoy an alternative dessert, without compromising taste, while managing their blood glucose. At the time of the manuscript preparation, the MM and SM cost USD 1.12/serving and USD 1.22/serving ($USD), respectively (−8% difference). It is also important to note that the SM flavor (Triple Chocolate Chunk) has been discontinued, whereas the MM flavor is still commercially available. Moreover, the cost per MM serving aligns with the affordability metrics stipulated in another international muffin market standard assessment [14]. This analysis thus revealed that the MM is (1) cost-effective and (2) effective for blood glucose management, making it a reasonable alternative to standard market products.

Several limitations need to be acknowledged to inform future study designs. The present sample size was small and largely comprised female participants (74%), owing to convenience sampling and the exploratory nature of the study design. Larger and more homogenous male and female cohorts are warranted in future investigations to precisely evaluate personalized nutrition. Two-thirds of adults with T2D in this study were prescribed glucose-lowering or insulin-based medications, which may have plausibly altered PPG responses to the product consumption. Since we did not standardize or ask participants to discontinue any medications prior to testing, we anticipated heterogeneity in glucose-lowering medications and accounted for these differences with a normalized data analysis approach. Plasma insulin responses were analyzed over three timepoints to characterize a predictable peak and iAUC that aligned with the prior methodology [26]. To characterize novel muffin kinetics more robustly, we recommend adding additional timepoints and metabolites to further highlight the postprandial differences between products.

## 5. Conclusions

In conclusion, consuming a low-sugar muffin product made with wheat-free grains confers improved postprandial glucose and insulin responses compared to a standard high-glycemic index, energy-matched muffin made with traditional refined wheat flour and sugar. Both products were also rated similarly in overall taste, flavor, and sensory evaluations. These features provide evidence to support that, without factoring in cost or availability, alternative low-sugar muffin products may replace standard high-GI muffin products without compromising taste/flavor and minimize postprandial glycemic effects commonly elicited by sucrose-sweetened products.

## Figures and Tables

**Table 1:** Muffin product nutritional characteristics.

Description	MM Product ^1^	SM Product ^2^	Net Difference (MM–SM)
Total Energy (kcal)	250	240	10
Carbohydrate (g)	27	30	−3
Sugar (g)	1	17	−16
Fiber (g)	5	3	2
Erythritol (g)	14	0	14
Fat (g)	12	13	−1
Saturated Fat (g)	2	4.5	−2.5
Trans Fat (g)	0	0	0
Protein (g)	6	4	2
Sodium (mg)	140	250	−110
Serving Size	2 Muffins (40 g)	1 Muffin (43 g)	−3
Commercial Product	TruEats (Arora Food Group)	Duncan Hines (Conagra Brands)	

^1^ TruEats (Arora Food Group) ingredients: erythritol, chickpea flour, Dutch cocoa powder, almond flour, buckwheat flour, moong dal flour, urad dal flour, baking soda, cream of tartar, sea salt, xanthan gum, sunflower lecithin, natural flavors, and monk fruit extract. ^2^ Duncan Hines (Conagra Brands) ingredients: flour (whole wheat flour and wheat flour), sugar, milk chocolate chunks (sugar, whole milk powder, cocoa butter chocolate liquor, butteroil, soy lecithin, salt, and natural flavor), chocolate chips (sugar, chocolate liquor, cocoa butter, soy lecithin, and vanilla extract), cocoa powder processed with alkali, palm oil, baking powder (monocalcium phosphate and baking soda), corn starch, dextrin (wheat fiber), salt, xanthan and guar gum, and natural flavor. The rows highlighted in grey denote the macronutrient categories.

**Table 2:** Participant characteristics.

Variable	Healthy Group (*n* = 10)			T2D Group (*n* = 9)			*t*-Test
	(3M:7F)			(2M:7F)			
	Mean	SD	Range	Mean	SD	Range	*p*-Value
Age (years)	39.2	7.1	30–50	39.4	5.1	30–46	0.954
Weight (kg)	78.0	15.6	55.4–102.7	107.2	22	70.8–152.9	**0.041**
BMI (kg/m^2^)	27.9	4.3	21.7–36.8	37.7	5.2	28.0–45.3	**0.003**
HbA1c (%)	4.8	0.4	4.4–5.7	5.7	0.6	4.7–6.6	**0.002**
FBG (mg/dL)	93.1	8.3	79–105	115.5	23.9	94–173	**0.012**
FPI (µIU/mL)	10.7	7.8	3.4–28.5	26.1	11.3	8.7–47.5	**0.005**
HOMA-IR	2.5	1.7	0.8–6.9	7.7	5.1	2.3–20.3	**0.006**

Values are mean ± SD. BMI, Body Mass Index; FBG, Fasting Blood Glucose; and FPI, Fasting Plasma Insulin. Bold values denote statistical significance (*p* < 0.05).

**Table 3:** Sensory evaluations (*n* = 19).

Variable	Rating Scale *	MM		SM		*t*-Test
		Mean	SD	Mean	SD	*p*-Value
Overall Liking	9	6.5	1.6	7.0	1.4	0.293
Flavor Liking	9	6.7	1.7	7.0	1.4	0.482
Mouthfeel Liking	9	7.1	1.5	7.1	1.4	1.000
Flavor Strength JAR	5	3.1	0.4	3.0	0.4	0.331
Sweetness JAR	5	2.9	0.5	3.1	0.5	0.331
Mouth-Drying Sensation JAR	5	3.3	0.5	3.4	0.5	0.429

Values are mean + SD. * The liking responses (top three rows) ranged from 1—dislike extremely—to 9—like extremely. The JAR responses (bottom three rows) ranged from 1—much too weak—to 5—much too strong.

## Data Availability

The raw data supporting the conclusions of this article will be made available by the authors on request.

## Supplementary Materials

The following supporting information can be downloaded at https://www.mdpi.com/article/10.3390/foods14244318/s1, Table S1: Modified Muffin (MM) Nutrition Facts, Ingredients, and Preparation Instructions; Table S2: Cooking Considerations; Table S3: Standard Muffin (SM) Nutrition Facts, Ingredients, and Preparation Instructions.

## Abbreviations

BMI	Body Mass Index
FBG	Fasting Blood Glucose
FPI	Fasting Plasma Insulin
GI	Glycemic Index
GLU_Peak_	Peak Normalized Glucose
GLU_P-N_	Peak–Nadir Glucose Amplitude
HbA1c	Glycated Hemoglobin
iAUC	Incremental Area Under the Curve
MM	Modified Muffin
PPG	Postprandial Glucose
SM	Standard Muffin
T2D	Type 2 Diabetes
ΔGLU	Peak Glucose Amplitude from Baseline
