# Supplementary material for: Postprandial Glycemia and Insulinemia Responses to a Standard and Modified Muffin in Healthy Adults and Adults with Type 2 Diabetes
Source: Foods. 2025 Dec 15;14(24):4318. doi: 10.3390/foods14244318 (PMC12733248; doi:10.3390/foods14244318)
Supplement: Supplementary file 1 [file foods-14-04318-s001.zip › foods-4017037-supplementary.pdf]

Supplementary File

Table S1. Modified Muffin (MM) Nutrition Facts, Ingredients, and Preparation Instructions

**Nutrient**	**Packaged (40g Mix)**	**Prepared (2 Muffins)**
**Calories**	170	250
Total Fat	4g 5%	12g 15%
Saturated Fat	1g 5%	2g 10%
Trans Fat	0g	0g
Cholesterol	0mg 0%	48mg 16%
Sodium	130mg 6%	140mg 6%
Total Carbohydrate	27g 10%	27g 10%
Dietary Fiber	5g 16%	5g 16%
Total Sugars	1g	1g
Added Sugars	0g 0%	0g 0%
Erythritol	14g	14g
Protein	5g 10%	6g 12%
Vitamin D	0mcg 0%	0mcg 0%
Calcium	40mg 3%	50mg 3%
Iron	5mg 28%	5mg 28%
Potassium	470mg 10%	490mg 10%

Additional Preparation Items

- Eggs → 4
- Oil → ½ cup
- Water → 1 cup (substitute with egg replacer for vegans)

Table S2. Cooking Considerations

PREHEAT oven according to the following:

Pan Type	Muffins Light	Muffins Dark	Cake Light	Cake Dark
Bake Temp	350°F	325°F	350°F	325°F
Bake Time	18–20 mins		28–32 mins	

PREP Spray muffin or cake pan with non-stick cooking spray or oil, or use paper cups.

COMBINE mix with eggs, water, and oil in a medium-sized bowl, and whisk until batter is smooth.

SPOON batter evenly into prepared muffin or cake pan.

BAKE in center of oven for recommended time or until center bounces back when pressed and toothpick comes out clean.

COOL completely before serving or frosting. Enjoy!

Ingredients: Erythritol, Chickpea Flour, Dutch Cocoa Powder, Almond Flour, Buckwheat Flour, Moong Dal Flour, Urad Dal Flour, Baking Soda, Cream of Tartar, Sea Salt, Xanthan Gum, Sunflower Lecithin, Natural Flavors, Monk Fruit Extract

Contains: Almonds

Table S3. Standard Muffin (SM) Nutrition Facts, Ingredients, and Preparation Instructions.

Nutrient	Per 1/12 dry mix (43g)		Per 1/12 baked muffin	
Calories	180		240	
	Amount	% DV*	Amount	% DV*
Total Fat	6g	8%	13g	17%
Saturated Fat	3.5g	18%	4.5g	23%
Trans Fat	0g		0g	
Cholesterol	0mg	0%	35mg	12%
Sodium	240mg	10%	250mg	11%
Total Carbohydrate	30g	11%	30g	11%
Dietary Fiber	3g	11%	3g	11%
Total Sugars	17g		17g	
Incl. Added Sugars	13g	26%	13g	26%
Protein	3g		4g	
Vitamin D	0mcg	0%	0mcg	0%
Calcium	110mg	8%	110mg	8%
Iron	2.4mg	15%	2.5mg	15%
Potassium	170mg	4%	190mg	4%

Additional Preparation Items:

- 2 large eggs
- ⅔ cup water
- ½ cup vegetable oil

DO NOT EAT RAW BATTER

PREP PREHEAT oven to 400°F. SPRAY muffin pans with PAM® or line with paper baking cups. RINSE and DRAIN blueberries thoroughly with cold water. Set aside.

MIX STIR muffin mix, water, eggs and oil together in a large bowl until moistened (about 50 strokes). Batter will be slightly lumpy. FOLD rinsed and drained blueberries gently into batter. SPOON batter into

muffin pans. Fill cups 2/3 full. SPRINKLE streusel topping evenly over each muffin and tap lightly into surface.

BAKE in center of oven following bake times provided. COOL 5 to 10 minutes. Gently loosen muffins before removing from pan.

Ingredients: Wheat Flour, Sugar, Milk Chocolate Chunks (Sugar, Whole Milk Powder, Cocoa Butter, Chocolate Liquor, Butteroil, Soya Lecithin, Salt, Natural Flavor), Chocolate Chips (Sugar, Chocolate Liquor, Cocoa Butter, Soy Lecithin, Vanilla Extract), Cocoa Powder Processed with Alkali, Palm Oil, White Wheat Bran, Wheat Germ, Leavening (Monocalcium Phosphate, Baking Soda), Corn Starch, Dextrin, Salt, Xanthan and Guar Gums+, Dextrose, Natural Flavor.
